# The combination of pre-neoadjuvant chemoradiotherapy inflammation biomarkers could be a prognostic marker for rectal cancer patients

**DOI:** 10.1038/s41598-022-07726-y

**Published:** 2022-03-11

**Authors:** Jing Zhang, Lin Zhang, Yuanyuan Gou, Panya Diao, Yi Hu

**Affiliations:** Department of Gastroenterology, Jiangjin District Central Hospital, Chongqing, China

**Keywords:** Cancer, Gastroenterology, Oncology

## Abstract

The neutrophil-to-lymphocyte ratio (NLR) and lymphocyte-to-monocyte ratio (LMR) have a strong association with prognosis in patients with Stage II/III rectal cancer (RC). We attempted to explore a new system combining these two ratios, named the NLM score, and examine its prognostic value in Stage II/III RC patients undergoing neoadjuvant chemoradiotherapy (NCRT). We retrospectively analyzed data of 237 stage II/III RC patients who underwent NCRT followed by standard TME in our hospital and defined the NLM score as follows: Score 2: pre-NCRT NLR > 2.565 and pre-NCRT LMR < 2.410. Score 1: (pre-NCRT NLR > 2.565 and pre-NCRT LMR > 2.410) OR (pre-NCRT NLR < 2.565 and pre-NCRT LMR < 2.410). Score 0: pre-NCRT NLR < 2.565 and pre-NCRT LMR > 2.410. Multivariate analyses implied that lower ypTNM stage (stage 0–I vs. II–III) (hazard ratio [HR] 0.420, 95% confidence interval [CI] 0.180–0.980 for OS; HR 0.375, 95% CI 0.163–0.862 for DFS) and an NLM score ≤ 1 (HR 0.288, 95% CI 0.134–0.619 for OS; HR 0.229, 95% CI 0.107–0.494 for DFS) could independently predict better overall survival (OS) and disease-free survival (DFS). The novel scoring system, which integrated pre-NCRT NLR and pre-NCRT LMR, was an independent prognostic factor in stage II/III RC patients undergoing NRCT and had better predictive values than these ratios alone.

## Introduction

Neoadjuvant chemoradiotherapy followed by radical resection is the standard treatment for stage III RC patients and stage II RC patients with high-risk factors^1,2^. While lymph node metastasis, vessel invasion, R1 or R2 resection, and high tumor stage are identified as unfavorable prognostic factors, patients with comparable risk elements have a wide variation of oncology outcomes^3,4^. This possibly contributes to the differences in the patients' tumor microenvironment and immune system^4,5^.

Evidence has shown that the systemic inflammatory response (SIR) is associated with cancer progression, evolution, and metastasis in recent years^6,7^. Neutrophil-to-lymphocyte (NLR) and Lymphocyte-to-Monocyte Ratio (LMR) are markers of SIR, and they are significantly related to the clinical prognosis of patients in various tumors^8–10^. Moreover, these markers are easy to obtain and inexpensive^9^. Nevertheless, all previous research focused on a single index, and the results of these studies were inconsistent. Consequently, we assumed that a scoring system combining these two ratios pre-NCRT might better prognostic prediction than the single ratio.

This study defined an innovative prognostic scoring system that integrated the pre-NCRT NLR and pre-NCRT LMR named NLM score and assessed its prognostic value for RC patients undergoing NCRT.

## Methods

### Patients population and data collection

We retrospectively reviewed the prospective clinical database of the Department of Gastroenterology, Jiangjin DistrictCentral Hospital. Stage II/III RC patients undergoing NCRT were included. The inclusion criteria were as follows: (1) RC patients undergoing 5-FU based NCRT. (2) Confirmed diagnosis of RC by biopsy (3) RC patients undergoing radical TME. The exclusion criteria were: (1) RC patients with distant metastasis. (2) Clinical indication of inflammatory disorder or infection, such as rheumatoid arthritis or inflammatory bowel disease; (3) Recurrent tumors (4) patients with other malignancies. (5) insufficient data.

Clinicopathological data were all obtained from electronic medical records, including age, gender, ypT stage, ypN stage, ypTNM stage, pathological CRM, vascular invasion, lymphatic invasion, perineural invasion, and laboratory data. Venous blood samples were drawn close to the time of NCRT initiation. Histopathological staging (ypT and ypN) was determined according to the American Joint Committee on Cancer Staging Manual (AJCC)^11^.

The Ethics Committee of Jiangjin District Central Hospital approved this study. Informed consent was waived by the Ethics Committee of Jiangjin District Central Hospital since it was a retrospective study.

All methods were performed in accordance with the relevant guidelines and regulations.

### Definition of pre-NCRT NLR, pre-NCRT LMR, and NLM score

We defined pre-NCRT NLR as a ratio of absolute neutrophil to lymphocyte counts., while pre-NCRT LMR as a ratio of absolute lymphocyte to monocyte counts.

The operating curve (ROC) analysis was adopted to determine the optimal pre-NCRT NLR and pre-NCRT LMR cut-off values for predicting death. The cut-off values were 2.565 and 2.410 for NLR and LMR, respectively. Same methods were performed for the optimal cut-off points for pre-NCRT CEA and CA19-9. The cut-off points for them were 3.55 and 19.0, respectively.

The NLM score was defined as: Score 2: NLR > 2.565 and LMR < 2.410. Score 1: NLR > 2.565 and LMR > 2.410 OR NLR < 2.565 and LMR < 2.410. Score 0: NLR < 2.565 and LMR > 2.410.

### Treatment, follow-up and endpoints

The multidisciplinary team meeting (MDT) conducted the treatment plan after considering recommendations of NCCN^12^, ESMO^13^, and JSCCR^14^ colorectal cancer guidelines and the patient's physical condition. All included patients received 5-fluorouracil-based chemoradiotherapy, followed by the standard total mesorectum excision (TME) procedure and pathological assessment of specimen^12–14^.

Patients were followed up every three months in the first three years after surgery and every six months after that. Blood tests were accomplished at each follow-up. Chest and abdominal CT scans were conducted every six months, with total colonoscopy one year after the operation and every two years afterward^12–14^. Clinical, radiological, or histological findings were used to monitor tumor recurrence^13^.

Follow-up data was gained by telephone or directly from outpatient clinic records^15^. Disease-free survival (DFS) and overall survival (OS) were primary endpoints^15^. OS was the time interval from operation date to death date or last visit, DFS is defined as the time interval from operation date to recurrence, metastasis, or last visit date^15,16^. Patients were censored if alive at the last follow-up^16^.

### Statistical analysis

Statistical analysis was performed by SPSS 22.0. Continuous and categorical variables were expressed as median ± interquarteral range and patients' numbers (%), respectively. Association between NLR or LMR and CEA and CA19-9 were assessed by Kaplan–Meier analysis with log-rank test was used for OS and DFS evaluation^17^.

Cox proportional hazard regression modeling was conducted for univariate (UV) and multivariate (MV) analyses^18^. UV analysis was performed to evaluate potential risk factors that could be associated with prognosis based on previous research and clinical knowledge^19^. Variables with a *p* value < 0.10 were included in MV regression analyses^20^. To avoid NLR and LMR's influence on the NLM score in the MV analysis, two models excluding and including the NLM score were established.

All statistical tests were bilateral, and 5% was set as the level of statistical significance^18–20^.

## Results

### Patients characteristics

We incorporated 237 patients according to the inclusion and exclusion criteria. The mean follow-up was 37 (range: 2–122) months. Thirty-six (15.2%) patients died during the follow-up. Among the 237 patients, 150 (63.3%) were male, and 87 (36.7%) were female. During pathological assessment after NCRT and operation, 45 (19.0%) patients presented ypTNM stage 0 (complete response), while 57 (24.1%) in ypTNM stage I, 72 (30.4%) in ypTNM stage II, and 63 (26.6%) in ypTNM stage III. Sixty (25.3%) patients presented lymph node metastasis, thirteen (5.5%) with vascular invasion, thirteen (5.5%) with lymphatic invasion, and forty-one (17.3%) with perineural invasion. The median (IQR) were 4.15 (2.18–10.07) for pre-NCRT CEA, 13.56 (7.80–25.40) for, 2.27 (1.77–2.98) and Detailed information was shown in Table 1.

**Table 1:** Clinicopathological characteristics for all included patients.

Characteristics	N (%)/median (IQR)
**Gender**	
Male	150 (63.3%)
Female	87 (36.7%)
**ypT stage**	
pT0–pT2	118 (49.8%)
pT3–pT4	119 (50.2%)
**ypN stage**	
pN0	117 (74.7%)
pN+	60 (25.3%)
**ypTNM stage**	
0	45 (19.0%)
I	57 (24.1%)
II	72 (30.4%)
III	63 (26.6%)
**Vascular invasion**^a^	
Absent	224 (94.5%)
Present	13 (5.5%)
**Lymphatic invasion**^a^	
Absent	224 (94.5%)
Present	13 (5.5%)
**Perineural invasion**^a^	
Absent	196 (82.7%)
Present	41 (17.3%)
**CRM**^a^	
Negative	229 (96.6%)
Positive	8 (3.4%)
Pre-NCRT CEA	4.15 (2.18–10.07)
Pre-NCRT CA-199	13.56 (7.80–25.40)
Pre-NCRT NLR	2.27 (1.77–2.98)
Pre-NCRT LMR	4.00 (2.95–5.11)
Age	57.00 (50.00–66.50)

NLR, neutrophil-to-lymphocyte ratio; LMR lymphocyte-to-monocytes ratio; NCRT, neoadjuvant chemoradiotherapy; Pre-NCRT NLR, NLR before patients receiving NCRT; Pre-NCRT LMR, LMR before patients receiving NCRT.

^a^Pathological status after neoadjuvant chemoradiotherapy.

### Associations between the pre-NCRT NLR, pre-NCRT LMR, and NLM score and prognosis

**Figure 1:**
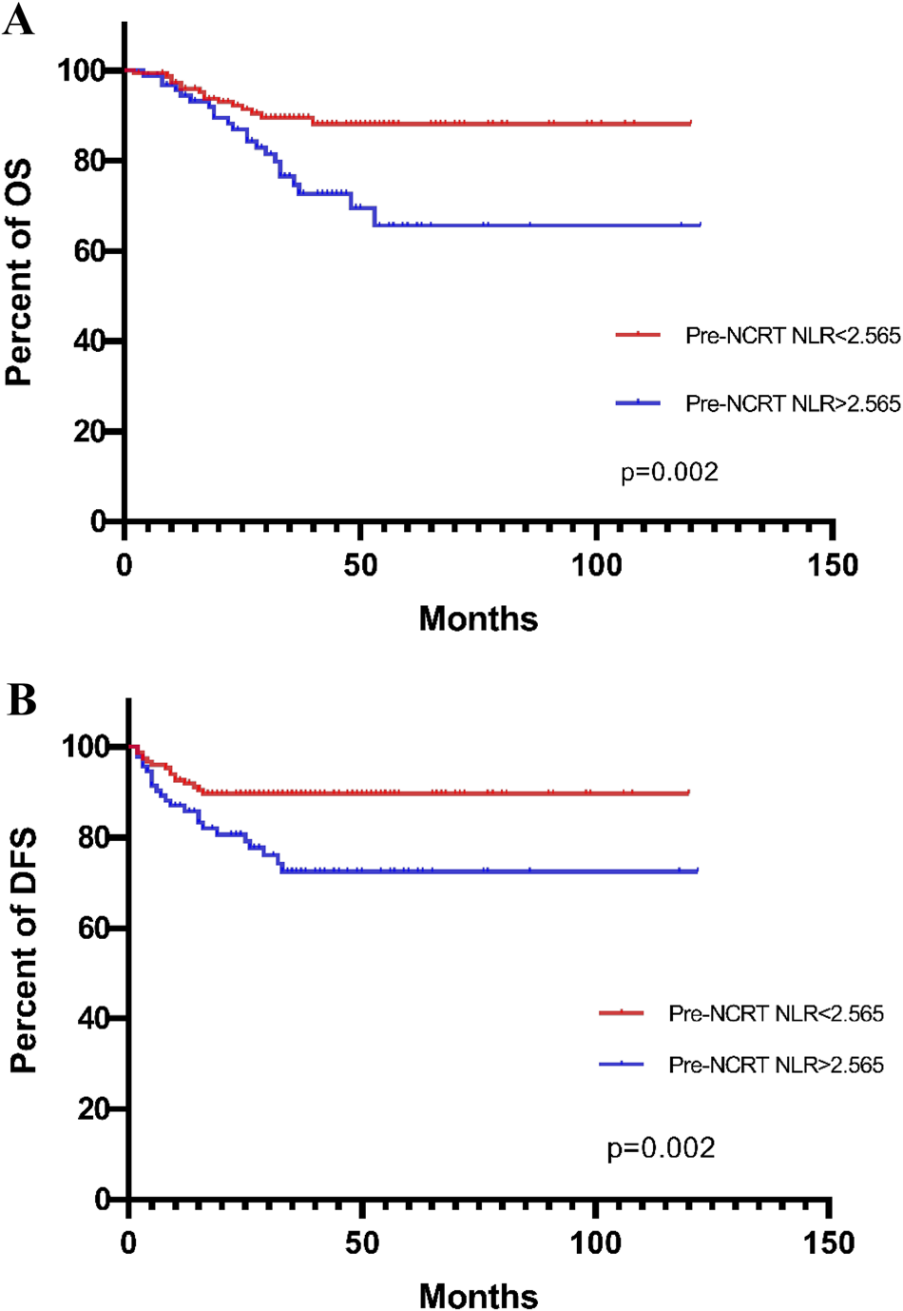
The pre-NCRT NLR > 2.565 had significantly worse OS and DFS than the pre-NCRT NLR < 2.565 (OS, *p* = 0.002, **A**; DFS, *p* = 0.002, **B**).

**Figure 2:**
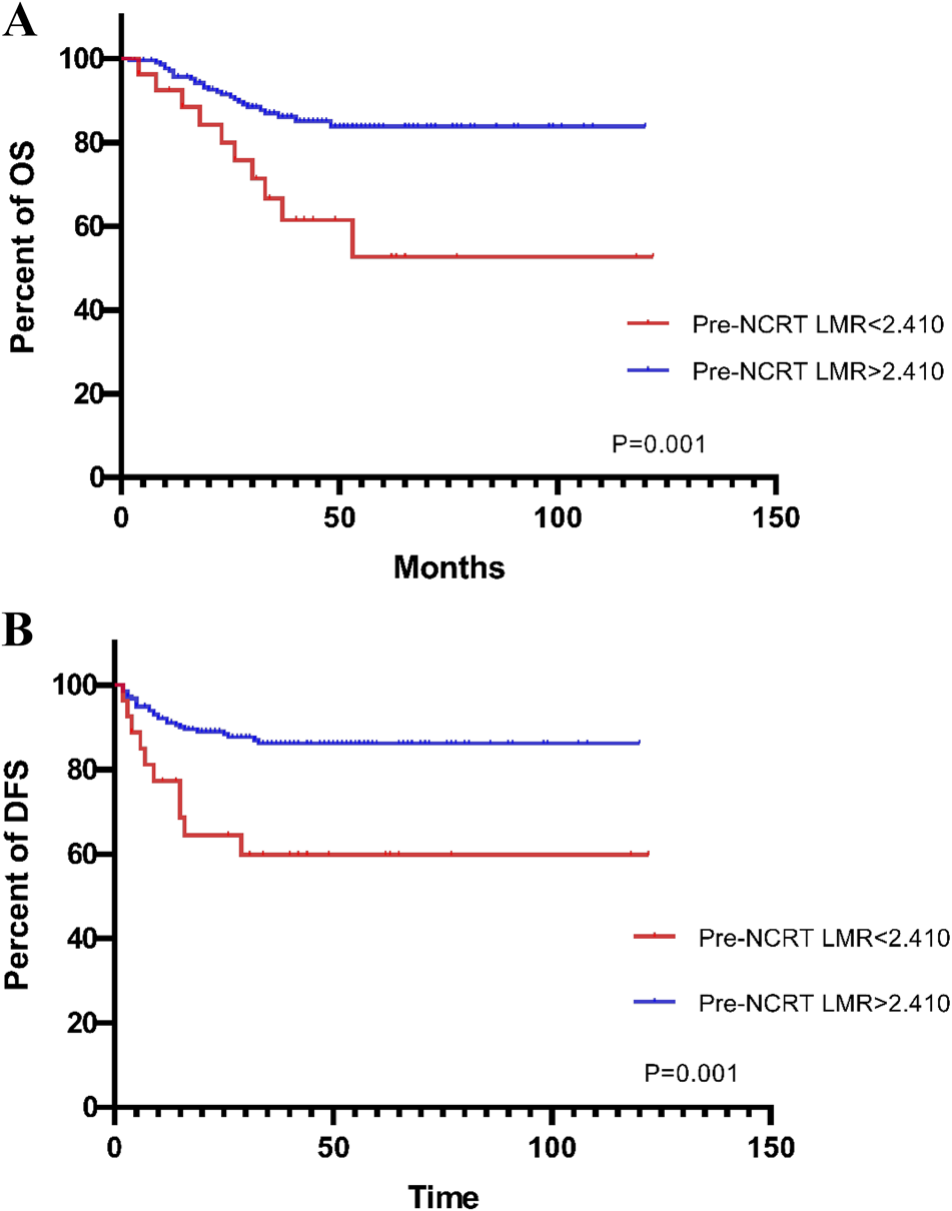
The pre-NCRT LMR < 2.410 had significantly worse OS and DFS than pre-NCRT LMR > 2.410 (OS, *p* = 0.001, **A**; DFS, *p* = 0.001, **B**).

**Figure 3:**
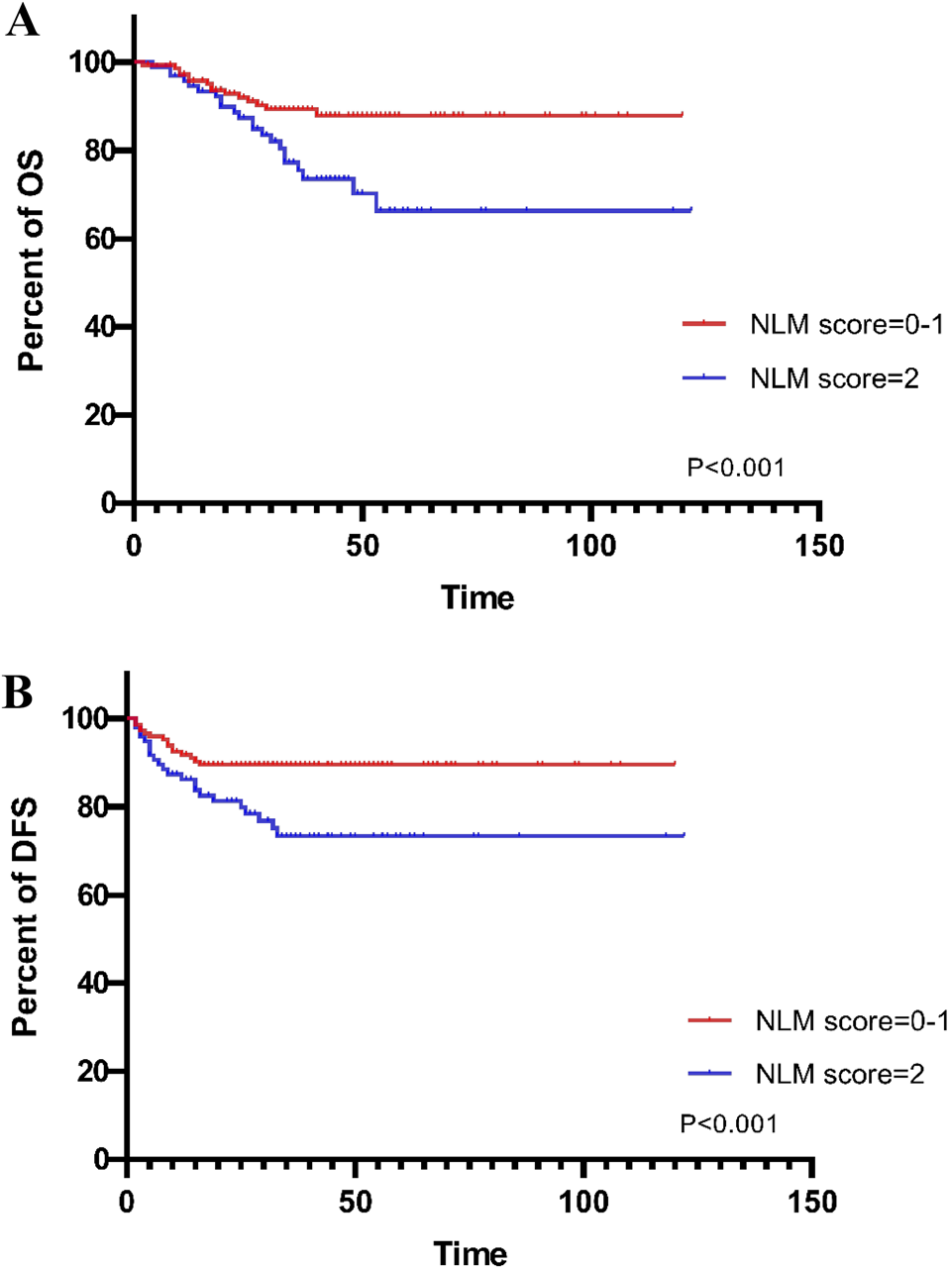
The higher NLM score predicted the worse OS and DFS (OS, **A**; DFS, **B**).

The pre-NCRT NLR > 2.565 had significantly worse OS and DFS than the pre-NCRT NLR < 2.565 (OS, *p* = 0.002, Fig. 1A; DFS, *p* = 0.002, Fig. 1B). The pre-NCRT LMR < 2.410 had significantly worse OS and DFS than pre-NCRT LMR > 2.410 (OS, *p* = 0.001, Fig. 2A; DFS, *p* = 0.001, Fig. 2B). The higher NLM score predicted the worse OS and DFS (OS, Fig. 3A; DFS, Fig. 3B).

### Univariate and multivariate analyses for DFS and OS

On univariate analysis, ypTNM stage, pre-NCRT CEA, pre-NCRT CA19-9, pre-NCRT NLR, pre-NCRT LMR and NLM score were significantly associated with OS (Table 2) and DFS (Table 3). MV analysis model 1 suggested that only ypTNM stage was an independent predictor for OS (HR 0.405, 95% CI 0.174–0.945, *p* = 0.037), while pre-NCRT NLR (HR 0.525, 95% CI 0.244–1.131, *p* = 0.100) and pre-NCRT LMR (HR 2.239, 95% CI 0.967–5.182, *p* = 0.060) were not. ypTNM (HR 0.353, 95% CI 0.154–0.811, *p* = 0.014) and pre-NCRT LMR (HR 2.739, 95% CI 1.190–6.304, *p* = 0.018) were significant predictors for DFS, while pre-NCRT NLR (HR 0.510, 95% CI 0.240–1.083, *p* = 0.510) was not. In MV analysis model 2, ypTNM (HR 0.420, 95% CI 0.180–0.980 for OS; HR 0.375 95% CI 0.163–0.862 for DFS) and NLM score (HR 0.288, 95% CI 0.134–0.619 for OS; HR 0.229, 95% CI 0.107–0.494 for DFS) were significant predictors for both OS and DFS.

**Table 2:** Univariate and multivariate analysis for OS.

Characteristics	Univariate analysis		Multivariate analysis			
	HR (95% CI)	*p* value	Model 1		Model 2	
			HR (95% CI)	*p* value	HR (95% CI)	*p* value
Gender (male/female)	2.091 (0.953–4.590)	0.066	1.633 (0.718–3.713)	0.242	1.669 (0.733–3.799)	0.222
ypTNM stage (0–I/II–III)	0.321 (0.141–0.732)	0.007	0.405 (0.174–0.945)	0.037	0.420 (0.180–0.980)	0.045
Vascular invasion (absent/present)	0.556 (0.170–1.821)	0.332	–	–	–	–
Lymphatic invasion (absent/present)	0.400 (0.141–1.136)	0.085	0.639 (0.208–1.959)	0.433	0.721 (0.241–2.157)	0.558
Perineural invasion (absent/present)	0.534 (0.250–1.141)	0.105	–	–	–	–
CRM (negative/positive)	0.826 (0.198–3.449)	0.826	–	–	–	–
Pre-NCRT CEA (< 3.55/> 3.55)	0.407 (0.185–0.893)	0.025	0.506 (0.214–1.195)	0.506	0.499 (0.212–1.173)	0.111
Pre-NCRT CA199 (< 19.0/> 19.0)	0.437 (0.225–0.849)	0.014	0.677 (0.322–1.422)	0.303	0.632 (0.304–1.311)	0.218
Pre-NCRT NLR (< 2.565/> 2.565)	0.355 (0.181–0.693)	0.002	0.525 (0.244–1.131)	0.100	–	–
Pre-NCRT LMR (< 2.410/> 2.410)	3.529 (1.702–7.319)	0.001	2.239 (0.967–5.182)	0.060	–	–
NLM score (0–1/2)	0.223 (0.108–0.464)	< 0.001	–	–	0.288 (0.134–0.619)	0.001

Model 1: including Pre-NCRT NLR and LMR into multivariate analysis, not including NLM score. Model 2: including NLM score into multivariate analysis, not including Pre-NCRT NLR and LMR. NLR, neutrophil-to-lymphocyte ratio; LMR lymphocyte-to-monocytes ratio; NCRT, neoadjuvant chemoradiotherapy; Pre-NCRT NLR, NLR before patients receiving NCRT; Pre-NCRT LMR, LMR before patients receiving NCRT; CI, confidence interval.

**Table 3:** Univariate and multivariate analysis for DFS.

Characteristics	Univariate analysis		Multivariate analysis			
	HR (95% CI)	*p* value	Model 1		Model 2	
			HR (95% CI)	*p* value	HR (95% CI)	*p* value
Gender (male/female)	2.144 (0.977–4.704)	0.057	1.797 (0.801–4.035)	0.155	1.762 (0.782–3.969)	0.172
ypTNM stage (0–I/II–III)	3.307 (1.448–7.549)	0.005	0.353 (0.154–0.811)	0.014	0.375 (0.163–0.862)	0.021
yp vascular invasion (absent/present)	0.571 (0.175–1.863)	0.353	–	–	–	–
yp lymphatic invasion (absent/present)	0.435 (0.154–1.231)	0.117	–	–	–	–
yp perineural invasion (absent/present)	0.595 (0.279–1.265)	0.177	–	–	–	–
yp CRM (negative/positive)	0.657 (0.158–2.738)	0.564	–	–	–	–
Pre-NCRT CEA (< 3.55/> 3.55)	0.391 (0.178–0.859)	0.019	0.424 (0.180–1.001)	0.050	0.433 (0.184–1.018)	0.055
Pre-NCRT CA199 (< 19.0/> 19.0)	0.413 (0.213–0.802)	0.009	0.693 (0.334–1.441)	0.326	0.638 (0.309–1.316)	0.224
Pre-NCRT NLR (< 2.565/> 2.565)	0.347 (0.178–0.679)	0.002	0.510 (0.240–1.083)	0.510	–	–
Pre-NCRT LMR (< 2.410/> 2.410)	3.954 (1.906–8.205)	0.001	2.739 (1.190–6.304)	0.018	–	–
NLM score (0–1/2)	0.192 (0.092–0.398)	< 0.001	–	–	0.229 (0.107–0.494)	< 0.001

Model 1: including Pre-NCRT NLR and LMR into multivariate analysis, not including NLM score. Model 2: including NLM score into multivariate analysis, not including Pre-NCRT NLR and LMR. NLR, neutrophil-to-lymphocyte ratio; LMR lymphocyte-to-monocytes ratio; NCRT, neoadjuvant chemoradiotherapy; Pre-NCRT NLR, NLR before patients receiving NCRT; Pre-NCRT LMR, LMR before patients receiving NCRT; CI, confidence interval.

### Relationship between NLR, LMR and CEA CA19-9 or other clinicopathological factors

Relationship between NLR, LMR and other clinicopathological factors were presented in Tables 4 and 5. Pre-NCRT NLR or LMR had no significant association between other factors.

**Table 4:** Relationship between pre-NCRT NLR or LMR and other clinic-pathological factors.

Characteristics	Pre-NCRT NLR < 2.565	Pre-NCRT NLR > 2.565	*p* value	Pre-NCRT LMR < 2.410	Pre-NCRT LMR > 2.410	*p* value
**Sex**			0.458			0.043
Male	91	59		20	130	
Female	57	30		4	83	
**ypTNM stage**			0.724			0.312
0–I	65	37		8	94	
II–III	83	52		16	119	
**Vascular invasion**			0.090			0.765
Absent	137	87		23	201	
Present	11	2		1	213	
**Lymphatic invasion**			0.269			0.519
Absent	138	86		22	202	
Present	10	3		2	11	
**Perineural invasion**			0.570			0.294
Absent	124	72		18	178	
Present	24	17		6	35	
**CRM**			0.457			0.335
Negative	142	87		24	205	
Positive	6	2		0	8	
Pre-NCRT CEA	4.02 (2.19–9.30)	4.64 (2.13–10.74)	0.614	3.38 (2.13–10.67)	4.23 (2.18–10.07)	0.793
Pre-NCRT CA199	12.61 (7.13–22.70)	15.95 (8.82–29.52)	0.053	15.66 (7.11–23.70)	13.38 (7.92–25.55)	0.903

**Table 5:** Association between pre-NCRT NLR or LMR and CEA or CA19-9.

Association factors	R	*p* value
CEA and NLR	− 0.003	0.967
CA199 and NLR	0.051	0.431
CEA and LMR	− 0.127	0.051
CA199 and LMR	− 0.075	0.251

## Discussion

Our research focused on the relationship between pre-NCRT NLR, pre-NCRT LMR, NLM score and OS and DFS in RC patients receiving NCRT. The main finding was that the NLM score was an independent prognostic factor in RC patients and was superior to pre-NCRT NLR and pre-NCRT LMR alone for predicting prognosis.

Previous studies explored the prognostic value of blood cell ratios for RC patients^21–23^. Shen et al.^24^ investigated 199 patients with locally advanced RC treated with NCRT followed by surgery. They presented a high NLR (≥ 2.8) independently related to poor OS in MV analysis but not to DFS^24^. The relationship between NLR and prognosis and the cut-off value was similar to ours, but their median follow-up period was relatively short (31 months). Carruthers et al.^25^ assessed 115 patients, showing that high NLR (≥ 5.0) was an independent prognostic factor for worse OS, decreased time to local recurrence, and shorter DFS^25^. Their results were consistent with ours in UV analysis. However, they only focused on the prognostic value of the single blood index, not comparing it to others nor combing it with others for better prognosis prediction.

Zhang et al.^26^ analyzed 472 LARC patients undergoing NCRT and radical surgery. They indicated a high NLR (> 2.3) was an independent predictor for OS and DFS in MV analysis, while LMR was only in UV analysis^26^. We found that NLR was an independent predictor for only DFS, while LMR was neither. Although we had similar results, they did not combine NLR and LMR for a more powerful prognostic factor. Our study presented that the NLM score had a better prognostic value than both NLR and LMR for both OS and DFS.

Although the mechanism of inflammation index affecting the prognosis of RC is not clear, some meaningful progress has been made. Tumor-associated neutrophils (TANs) origin from peripheral neutrophils^27,28^. They play an essential role in tumor progression since they could promote tumor growth, cause genetic instability and stimulate angiogenesis^27,28^. Tumor-associated macrophages (TAMs) are derived from circulating monocytic precursors and are essential in tumor progression's inflammatory microenvironment^29^. TAMs can produce angiogenesis and growth factors and protease enzymes, which promote extracellular matrix degradation, angiogenesis, tumor cell proliferation, and metastasis^30^. Different from monocytes and neutrophils, lymphocytes is essential in host cell-mediated immune regulation, which helps destroy residual malignant cells and related micrometastases^29^. Temporarily, tumor-infiltrating lymphocytes are related to improving the clinical prognosis of cancer^27–30^. All of the mechanisms we examined may explain why patients with a high NLR or a low LMR have poor prognosis.

Although similar studies focused on the prognostic value of immune indexes, they only focused on single factors and did not integrate these biomarkers nor compare their prognostic values^8,21,22,25,26,29^. This study established a novel scoring system combining pre-NCRT NLR and pre-NCRT LMR, named NLM score. The NLM score was superior to single ratios for predicting RC prognosis. Our research found that pre-NCRT NLR has a better prognosis prediction effect than pre-NCRT LMR, indicating neutrophils and monocyte could have different effects on tumor micrometastasis. More profound research in this area may be valuable.

This study had several limitations. Firstly, it was a retrospective study conducted at a single center. Secondly, in this analysis, the optimal cut-off points for pre-NCRT NLR and pre-NCRT LMR were 2.565 and 2.410, respectively. Nevertheless, the cut-off point in this study may only apply to our center's population. If doctors from other medical centers try to apply this prognostic scoring system, we recommend conducting their analysis to determine the optimal cut-off point for a particular patient group. Thirdly, we only enrolled the pre-NCRT NLR and pre-NCRT LMR into our prognostic scoring system. Other systemic inflammatory biomarkers still have high prognostic significance, such as the prognostic nutritional index.22,33 Regrettably, they were not available regularly in our department. Future research on the prognostic scoring system should include as many systemic inflammation indices as possible.

## Conclusion

In conclusion, we established a novel prognostic system combing pre-NCRT NLR and pre-NCRT LMR for RC patients. We demonstrated an NLM score ≤ 1 could independently predict better survival. Further studies are necessary to verify its prognostic value.
